# The Transportation of Wounded Men in Motor Ambulance Convoys

**Published:** 1918-09

**Authors:** G. Poe


**The Transportation of Wounded Men from the Point of View of Motor Ambulance Convoys.** Colonel G. Poe., R. A. M. C.

*Motor ambulance convoy*, the speaker said, is classed as "Army troops", allotted usually at the rate of one Motor ambulance convoy to a corps. A Convoy is composed as follows :

| | | |
|---|---|---|
| Officers R. A. M. C. | | 1 |
| " A. S. C. (Road Officer) | | 1 |
| " " | (Workshop) | 1 |

| | |
|---|---|
| Other Ranks R. A. M. C. | 18 |
| " " A. S. C. (Drivers et Workshop) | 104 |
| Motor Ambulances | 50 |
| Touring Cars | 2 |
| Motor Cycles | 7 |
| Motor Lorries (Workshop) | 1 |
| " " (Supply) | 1 |
| " " (Stores) | 2 |

The commanding officer has no professional work to do as none is practicable.

The usual function of a motor ambulance convoy is to clear sick and wounded from the main dressing station to the casualty clearing station. The commanding officer may, occasionally, be ordered to lend cars to a division to clear casualties from the advanced dressing station to the main dressing station if the divisional cars are insufficient.

Pooling of all divisional cars and motor ambulance convoy cars has been tried but the experiment was not always a success.

Cars should be heated by the extension of the exhaust; the pipes from which are carried along in front of each seat.

Each car should carry hot-water bottles, 4 stretchers, and 12 blankets.

A trained R. A. M. C. orderly is required in each car carrying serious head injuries, or cases of hemorrhage.

The chief point is to ensure a continuous flow of cars.

Taking a corps in action with two divisions in the line and two main dressing stations at " A " and " B ", a suitable place " C " for parking cars should be selected on a common route from " A " and " B " to the casualty clearing station. Five or six cars — *not more* — should be sent to " A " and " B " at the commencement of the action. As a car passes " C ", loaded, and on its way to the casualty clearing station, an empty car should forthwith move up to whichever main dressing station the loaded car came from. On return from the casualty clearing station, empty, all cars should go to " C " and take their places on the stand. There will usually be time for the driver to have some food and a rest before again moving up to the main dressing station.

The number of cars required depends (1) on the distance to the casualty clearing station; (2) the state of the roads; (3) the amount of congestion of the traffic on the roads. Cars frequently take five hours to complete the double journey. If the divisional main dressing station can fill a car every twenty minutes, this would mean seventeen cars on the circuit, otherwise an accumulation of wounded would take places at the main dressing station.

In ordinary "peace warfare", that is, when no definite battle is in progress, the convoy cars take sitting or lying cases indiscriminately to the casualty clearing station.

During a battle the convoy cars only take stretcher cases and bad sitting cases.

Walking wounded are usually helped along by motor lorries and horsed ambulances to a central point, the corps walking wounded collecting post, from whence they are taken by lorry, light railway, or broad gauge railway to a separate casualty clearing station.

At the corps walking wounded post many cases who have struggled along that far, either by lorry or by walking, become unfit to proceed further by lorry. A certain number of motor ambulance cars — probably five or six — should be kept available for carrying such lying down cases to the casualty clearing station from the corps walking wounded post.

Motor ambulance convoys held in reserve by armies, or corps motor ambulance convoys are also sometimes used for evacuating cases from one casualty clearing station to another further back, when no ambulance trains are available.

This is the use made of a motor ambulance convoy in the British Army, chiefly to keep up a circuit of cars from main dressing stations to casualty clearing stations. If working smoothly, no accumulation of waiting cars or waiting patients should occur at a main dressing station.

The circuit of cars between the advanced dressing station and the main dressing station is done by the motor ambulances belonging to the field ambulances of the division; these are grouped together and used as described by Colonel Ensor.

The great point is to avoid a large group of cars at the main dressing station.

At the main dressing station, the ideal to be aimed at, as far as motor ambulances are concerned, is to have a separate road of entry and exit: (1) for divisional cars, and (2) for motor convoy cars. Separate squads of bearers meet the divisional cars, unload them, and carry the wounded into the receiving room; other squads wait in the evacuation room, carry the patients out, and load the motor ambulance convoy cars. At the casualty clearing station, bearers should be ready to unload the motor ambulance convoy cars immediately, and to hand over to it a number of hot water bottles, stretchers, and blankets equivalent to those received with the patients.

